# Silibinin-Loaded Amphiphilic PLGA–Poloxamer Nanoparticles: Physicochemical Characterization, Release Kinetics, and Bioactivity Evaluation in Lung Cancer Cells

**DOI:** 10.3390/ma17225480

**Published:** 2024-11-10

**Authors:** Fabrizio Villapiano, Miriam Piccioni, Federica D’Aria, Stefania Crispi, Giovanna Rassu, Paolo Giunchedi, Elisabetta Gavini, Concetta Giancola, Carla Serri, Marco Biondi, Laura Mayol

**Affiliations:** 1 Department of Pharmacy, University of Naples Federico II, Via Domenico Motesano 49, 80131 Naples, Italy; fabrizio.villapiano@unina.it (F.V.); federica.daria@unina.it (F.D.); giancola@unina.it (C.G.); 2 Institute of Biosciences and Bio-Resources, National Research Council (CNR-IBBR), 80100 Naples, Italy; miriam.piccioni@ibbr.cnr.it (M.P.); stefania.crispi@ibbr.cnr.it (S.C.); 3 Department of Medicine, Surgery and Pharmacy, University of Sassari, Via Muroni 23/A, 07100 Sassari, Italy; grassu@uniss.it (G.R.); pgiunc@uniss.it (P.G.); eligav@uniss.it (E.G.); 4 Department of Advanced Biomedical Sciences, University of Naples Federico II, Via Pansini, 80131 Naples, Italy; laura.mayol@unina.it

**Keywords:** silibinin, stealth nanoparticles, poloxamer/PLGA nanoparticle stability, lung cancer cells

## Abstract

Despite its potential against several carcinomas, the pharmacological efficacy of silibinin (SLB) is hampered by poor solubility, absorption, and oral bioavailability. To face these issues, we developed polylactic-co-glycolic acid (PLGA) nanoparticles (NPs) coated with hydrophilic polyethene oxide (PEO) for controlled and targeted SLB delivery. NPs were produced at two different SLB loadings and presented a spherical shape with smooth surfaces and stable size in water and cell culture medium. The encapsulation efficiencies were found to be >84%, and thermal analysis revealed that the SLB was present in an amorphous state within the NPs. In vitro SLB release experiments revealed that at the lowest SLB loading, desorption of the active molecule from the surface or nanoporosities of the NPs mainly dictates release. In contrast, at the highest SLB loading, diffusion primarily regulates release, with negligible contributions from other mechanisms. Cell experiments showed that, compared with the free drug, SLB loaded in the produced NPs significantly increased the bioactivity against H1299, H1975, and H358 cells.

## 1. Introduction

**Figure 1:**
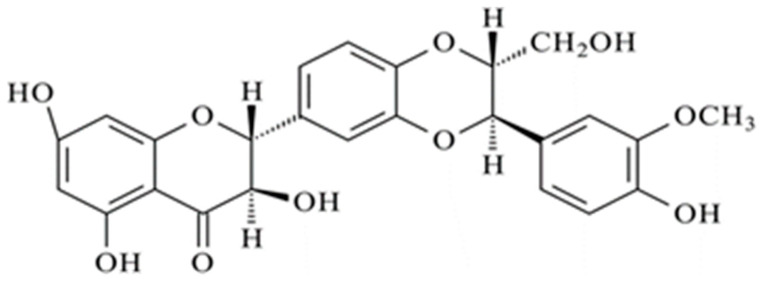
Structure of silibinin.

Silymarin is a flavonoid derived from the seeds of the medicinal plant *Silybum marianum*, commonly known as milk thistle, and has been widely used in the medical field, especially for the treatment of liver disease (Figure 1). Silymarin is composed of seven flavonolignans (silibinin, isosilibinin, silycristin, isosilycristin, and silydianin) and one flavonoid (taxifolin) [1]. In particular, silibinin (SLB) is the biologically active compound of silymarin and is composed of two diastereoisomers (silybin A and silybin B) [2,3]. Recent studies have shown that SLB possesses inhibitory effects on multiple cancers, such as prostate, colon, breast, and skin cancer [4]. SLB also possesses significant antiproliferative activity and has effects on the induction of apoptosis, chemosensitization, growth inhibition, reversal of multidrug resistance (MDR), and inhibition of angiogenesis, tumor invasion, and metastasis [5,6,7,8]. Nonetheless, the efficacy of SLB is drastically hindered by its low aqueous solubility (<0.1 mg/mL) and poor bioavailability after oral administration [9,10], which causes the need for high doses to elicit adequate plasma levels. To overcome this issue, a wide array of delivery systems have been developed to increase SLB solubility and bioavailability [11]. To this end, the formulation and use of polymeric nanoparticles (NPs) for drug delivery are promising for the treatment of cancer compared to traditional chemotherapy [12]. Moreover, NPs endowed with a hydrophilic surface possess prolonged circulation properties which in turn allow them to take advantage of the enhanced permeability and retention (EPR) effect of solid tumors [13]. Such NPs can extravasate within tumors through aberrant vasculature resulting from uncontrolled angiogenesis [13,14,15,16,17,18] or inflamed or compromised capillary endothelium, and be retained for prolonged periods of time due to the reduced draining of fluids from the tumor [12]. Among the polymers used to produce NPs for tumor treatment, poly(lactic-co-glycolic acid) (PLGA) is a material of choice. Indeed, PLGA NPs have several advantageous properties [19], such as biocompatibility, biodegradability, and the possibility of obtaining targeted drug delivery [13,20]. These are pivotal features in that they allow the minimization of the systemic toxicity associated with traditional chemotherapy. The functionalization of these carriers with specific ligands can further improve their specificity toward tumor tissues. PLGA particles are quickly phagocytosed after intravenous injection depending on their size, thereby attaining tendentially short circulation times [20]. This problem can be attenuated by preparing NPs using a blend of PLGA and poloxamers. In previous works, we produced NPs based on a physical blend of PLGA and poloxamers F127 and F68 to confer stealth properties to the produced NPs [13,16]. Specifically, poloxamers are amphiphilic triblock copolymers consisting of poly(ethylene oxide)–poly(propylene oxide)–poly(ethylene oxide) (PEO–PPO–PEO) segments and, when added to NPs produced by single/double emulsion or nanoprecipitation techniques, spontaneously arrange the hydrophilic ethylene oxide segments toward the external aqueous phase, thus making the surface hydrophilic and conferring stealth properties to the NPs. Stealth NPs are designed to evade immune detection, employing polyethylene glycol (PEG) or poloxamers for improved circulation. PEG forms a hydrated steric barrier around NPs, reducing aggregation and prolonging systemic presence [13,21]. Poloxamers similarly utilize EO for creating a hydrated protective layer. The unique triblock configuration of poloxamers allows the hydrophobic PO block to anchor effectively to NP surfaces, potentially enhancing stability in complex biological environments. While PEG provides uniform surface coverage, poloxamers offer robust anchoring due to their amphiphilic nature [13,22,23]. In our previous studies, NPs based on physical blends of PLGA–poloxamers were shown to increase the stability of the produced devices in cell culture media [20] and promote their accumulation in vivo in the lung in a murine model [20]. Furthermore, the addition of poloxamers has been shown to increase the circulation time of NPs after intravenous administration [13,14,16,17,24,25,26], thus providing NPs with passive targeting ability [27]. Therefore, the objective of this work was to load SLB within PLGA–poloxamer NPs and assess their bioactivity against different lung tumor cells. To this aim, NPs were produced by the nanoprecpitation technique and fully characterized for their technological and thermodynamic features, along with their release mechanism. Also, the in vitro bioactivity of the produced NPs was assessed against three human lung cancer cell lines, namely H1299, H1975, and H358 cells [13,14,17].

## 2. Materials and Methods

### 2.1. Materials

Silibinin (SLB), 2,3-dihydro-3-(4-hydroxy-3-methoxyphenyl)-2-(hydroxymethyl)-6-(3,5,7-trihydroxy-4-oxobenzopyran-2-yl) benzodioxin, silybin, and equimolar uncapped poly(D,L-lactide-coglycolide) (PLGA) (Resomer RG504H, Mw 40 kDa) were purchased from Sigma-Aldrich (St. Louis, MO, Milan, Italy). Poloxamers F127 (a = 100 and b = 65) and F68 (a = 76 and b = 29) were obtained from Lutrol (Basf, Ludwigshafen, Germany). Potassium chloride (KCl) from Carlo Erba (Cornaredo, Italy) was used. Dimethylformamide (DMF), dimethyl sulfoxide (DMSO), ethanol (EtOH), acetone, dibasic sodium phosphate (Na2HPO4), and sodium chloride (NaCl) were obtained from J-Baker (Chicago, IL, USA). For cell culture experiments, Roswell Park Memorial Institute (RPMI-1640) medium supplemented with fetal bovine serum (FBS), 50 UI/mL penicillin, 0.05 mg/mL streptomycin, sodium pyruvate, and 4-(2-hydroxyethyl)-1-piperazineethanesulfonic acid (HEPES) from Euroclone (Rome, Italy) were used. All chemicals and media were used as received without any further purification. The concentration of the SLB stock solution was 150 mM in DMSO.

### 2.2. Preparation of Nanoparticles (NPs)

NPs were produced by the nanoprecipitation–solvent evaporation technique [13]. In brief, 5 mL of an oil phase composed of a PLGA or PLGA–poloxamer (PLGA:F68:F127, 2:1:1 weight ratio) solution (2% *w*/*v* polymers) were combined with 5 or 10 mg of SLB by vortexing for 10 min, as reported in Table 1. The obtained solution was then precipitated through a syringe needle (22 G) in 40 mL of an aqueous phase, containing F127 and F68 as surfactants (1:1 wt ratio; 0.375 mg/mL overall concentration), by a syringe pump (flow rate = 333.3 µL/min; d = 11.99 mm). After acetone evaporation by overnight stirring at room temperature, the obtained NP suspension was washed three times at 13,000 rpm for 10 min by centrifugation (Hettich Zentri-Fugen, Tuttlingen, Germany). Finally, NPs were stored at 4 °C.

### 2.3. Physicochemical Analysis of the NPs

#### 2.3.1. Particle Size and Surface Charge Analyses

The mean size, size distribution, and zeta potential of the produced NPs were attained by photon correlation spectroscopy (PCS; N5 Submicron Particle Size Analyser, Beckman Coulter, Miami, FL, USA, Beck-man-Coulter, and ζ-potential (ZP)) by dynamic light scattering (DLS) analyses carried out with a Zetasizer Ultra apparatus (Malvern Instruments, Malvern, UK) at room temperature. To perform the measurements, NPs were suspended in ultrapure water at a concentration of 0.1 mg/mL. Each sample underwent twelve runs at room temperature.

#### 2.3.2. Stability Study

The stability of the NPs was evaluated by tracking their size over time in an aqueous suspension at 4 °C, as well as in cell culture medium at 37 °C. The time trends of hydrodynamic diameters of unloaded, PP-SLB5, and PP-SLB10 NPs were tracked for up to 30 days in double-distilled water (storage conditions) and in RPMI-1640 medium supplemented with 10% FBS at 37 °C for up to 72 h. Additionally, NP size measurements were conducted on cell culture medium alone to check for any potential self-aggregation. The results presented were averaged from at least five individual measurements.

#### 2.3.3. Nanoparticle Morphology

Transmission electron microscopy (TEM) images of NPs were characterized using a TEM, TECNAI-12, FEI, Hillsboro, OR, USA. For this analysis, 100 μL of ultradiluted NP suspensions in water was placed onto a copper TEM grid (300 mesh, 3 mm in diameter).

#### 2.3.4. Yield, Drug Entrapment Efficiency, and Drug Loading of Nanoparticles

The yield of the NPs and SLB entrapment efficiency (η) and drug loading (λ) were determined from preliminarily freeze-dried NPs (0.01 atm, 24 h; Modulyo, Edwards, UK). Specifically, the NP yield was calculated based on the actual mass of the recovered freeze-dried NPs. For entrapment efficiency and drug loading tests, 100 μL of PP-SLB5 or PP-SLB10 NPs was mixed with 900 µL of DMSO and gently stirred for 30 min at room temperature to dissolve the particles and allow total SLB dissolution in the medium. The obtained solution was centrifuged for 15 min at 13,000 rpm, and SLB was quantified by a spectrophotometric assay (UV-1800, Shimadzu Laboratory World, Kyoto, Japan) at λ = 289.0 nm. The linearity of the response was verified over the concentration range of 0.2–50 μg/mL (y = 0.0367x − 0.0023; R^2^ > 0.999) [10,28]. η and λ were expressed as follows:

(1) $$\eta =100\cdot \frac{m({\mathrm{SLB}}_{\mathrm{entrapped}})}{m({\mathrm{SLB}}_{\mathrm{total}})}\;\lambda =100\cdot \frac{{\left[m(SLB\right)}_{total}-m({SLB)}_{entrapped}]}{\left[m\left(\mathrm{NPs}\right)+m\left({SLB}_{total}\right)\right]}$$

± the standard deviation (SD) calculated on three separate batches.

#### 2.3.5. Thermal Characterization via Differential Scanning Calorimetry (DSC)

Thermal analyses were performed by differential scanning calorimetry (DSC) on pure SLB and lyophilized SLB-loaded NPs to investigate drug–polymer and polymer–polymer interactions. Tests were carried out on powders of pure SLB and freeze-dried PP, PP-SLB5, and PP-SLB10 powders (24 h, 0.01 atm, −50 °C; Buchi, Flawil, Switzerland). The samples were inserted in aluminum pans and analyzed using a 10–250 °C temperature with a heating rate of 10 °C/min. The measurements were conducted in an inert nitrogen atmosphere purged at a 50.0 mL/min flow rate. The heat evolved during the thermic event (W/g) was calculated from the recorded DSC thermograms by integrating the exothermic/endothermic peaks, while the glass transition temperature (Tg) was obtained from the thermogram inflection point.

### 2.4. In Vitro Drug Release Studies

The release of SLB from the NPs was assessed by loading 4 mL of a NP suspension (at 14.56 mg/mL for PP-SLB5 and 19.33 mg/mL for PP-SLB10) into a dialysis membrane (Spectra/Por^®^ Biotech Cellular ester, Fisher Scientific, Bishop Meadow Road, Loughborough, Leicestershire, UK; molecular cut-off 12 kDa), which was immersed at 80 rpm in an orbital incubator (SI50, Stuart R, London, UK) containing phosphate buffer solution (PBS) 90% *v*/*v* or DMF 10% *v*/*v*, with the pH adjusted to 7.4. At predetermined time points, 1 mL aliquots of the release medium were taken and replaced with an equivalent volume of fresh medium. SLB was quantified by a spectrophotometric assay (UV-1800, Shimadzu, Kyoto, Japan) at a wavelength of 324.5 nm [10,28]. The instrument response was linear over the 0.1–50 μg/mL concentration range (y = 0.0358x + 0.0454; R^2^ > 0.997). The experiments were run in triplicate. In this study, we compared the results of various models to interpret release data, aiming to select the most suitable one for SLB release from formulated NPs [29]. The models are reported in the following:

The zero-order equation is as follows:

(2) $$F=K_{0}t$$

where F is the released drug fraction at time t and k0 is the zero-order release rate constant [30].

The first-order equation is as follows:

(3) $$In\left(1-F\right)=-k_{1}t$$

where k_1_ is the first-order release rate constant [31].

Higuchi’s equation is as follows:

(4) $$F=k_{H}t^{0.5}$$

where k_H_ is the Higuchi release rate constant [32].

The Korsmeyer–Peppas semiempirical model is as follows:

(5) $$In\left(F\right)=In\left(K_{KP}\right)+nIn(t)$$

where k_KP_ is the Korsmeyer–Peppas constant, which describes the structural and geometric characteristics of the device, and n is the release exponent, which indicates the main drug release. Each model was fit to average release data.

### 2.5. Cell Culture

The human lung cancer cell lines NCI-H1299 (ATCC CRL-5803), NCI-H1975 (ATCC CRL-5908), and NCI-H358 (ATCC CRL-358) were obtained from the ATCC (Manassas, VA, USA). The cells were routinely propagated in RPMI-1640 medium supplemented with 10% heat-inactivated fetal bovine serum (FBS), 1% L-glutamine, 1% sodium pyruvate, 50 UI/mL penicillin, and 0.05 mg/mL streptomycin (Euroclone, Milan, Italy). The cells were maintained at 37 °C in a humidified atmosphere containing 5% CO_2_ and were in the logarithmic growth phase at the start of the experiments. Subculturing was performed twice every week, beginning with a low-passage cell stock, for a period of 2–3 months following thawing. The cell lines were routinely tested for mycoplasma contamination using the MycoAlert Mycoplasma Detection Kit (Lonza, Verviers, Belgium).

### 2.6. In Vitro Bioactivity of PP-SLB Nanoparticles

For the cell proliferation assay and for each cell line, 3 × 10^4^ cells/well in 1 mL of growth medium were seeded in 12-well plates. Sixteen hours after seeding, the cells were treated with different concentrations of SLB alone or encapsulated in PP-SLB5 NPs. Before cell treatment with PP-SLB5 NPs, the formulation was filtered twice through 0.45 µm filters (filter size: 25 mm; AlfaTech, Genova, Italy) for enhanced sterility assurance. Cells treated with vehicle (DMSO) or unloaded NPs were used as controls. After 24 h of treatment, the cells were collected and counted with Trypan blue solution (Sigma-Aldrich, Merck KGaA group, Darmstadt, Germany). Cell viability was assessed by counting live cells using an MTS assay (CellTiter 96; Promega Corporation, Madison, WI, USA) following the manufacturer’s instructions. For the MTS assay, the absorbance was measured on a microplate reader at a wavelength of 490 nm (VICTOR Multilabel Plate Reader; PerkinElmer, Inc., Waltham, MA, USA). Cytotoxicity was expressed as a percentage compared to the control cells. The 50% inhibition concentration (IC50) was calculated from the growth curves using GraphPad Prism 10. All experiments were conducted in triplicate, and the results are presented as the mean ± standard deviation (SD).

### 2.7. Statistical Analysis

The statistical data analysis was conducted using GraphPad Prism 10 software (GraphPad Software, Inc., San Diego, CA, USA). An unpaired t test was utilized to identify significant differences between the two treatment groups. For multiple comparisons, Tukey’s multiple comparison tests followed by one-way ANOVA were used. Statistical significance was set at *p* < 0.0001 and *p* < 0.05.

## 3. Results and Discussion

The pharmacological potential of SLB is severely restricted by its extensive first-pass metabolism in the liver, thus resulting in an incomplete or irrelevant intestinal absorption, mainly due to its low aqueous solubility [33]. To face these solubility and bioavailability issues, we propose loading SLB in PLGA-based nanoparticles (NPs), both with and without poloxamers (PP and P formulations, respectively) [13] to attain controlled and targeted SLB delivery to the lung. In an earlier study we demonstrated that, after a single intraperitoneal injection, PP NPs were sequestered from the lungs, whereas P NPs were not detected in any organ [20]. We hypothesized that the incorporation of amphiphilic poloxamers into the organic phase used for NP production led to the spontaneous organization of hydrophilic ethylene oxide units toward the NP surface, thus providing the NPs with a hydrophilic surface that may change their pharmacokinetics, thereby promoting passive lung targeting.

### 3.1. Morphology and Characterization of NPs

**Figure 2:**
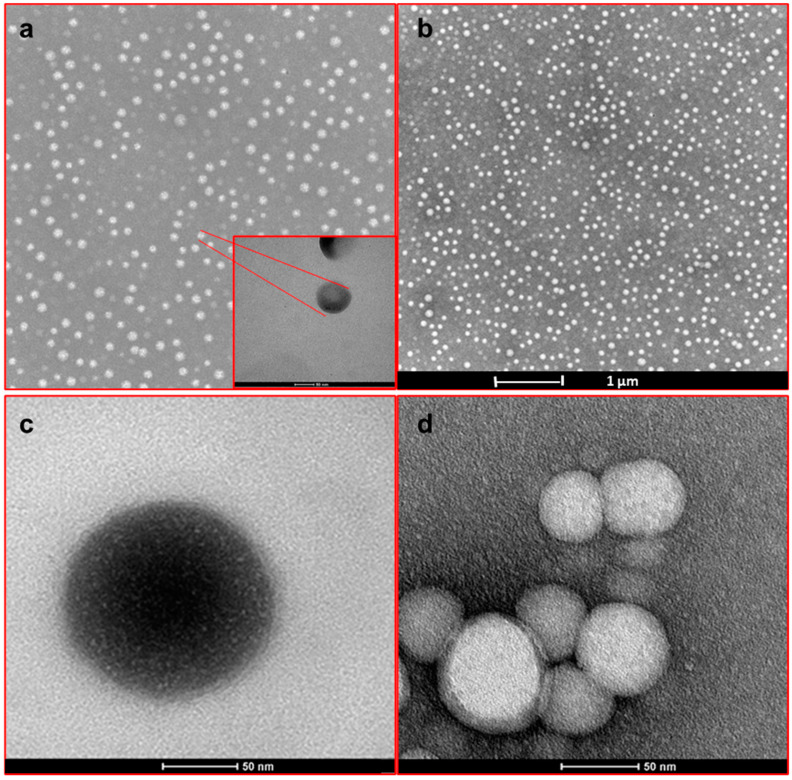
Representative TEM images at different magnifications of SLB-loaded PP NPs (**a**–**d**).

The results of TEM observations show that NPs are spherical with a regular surface (Figure 2) [13]. The sizes of the unloaded P and PPNPs were around 150 and 90 nm, respectively; the SLB-loaded NPs were around 100 and 124 nm for the PP-SLB5 and PP-SLB10 NPs, respectively (Table 2). Notably, SLB loading in NPs increased their mean diameter compared to unloaded NPs (*p* < 0.05), likely due to physical interactions of SLB with the poloxamers and PLGA, as previously demonstrated [9]. The polydispersity index (PDI) of PP NPs (0.132 ± 0.01) was significantly lower than those of PP-SLB5 and PP-SLB10 NPs (0.212 ± 0.03 and 0.302 ± 0.06, respectively) (Table 2). The inclusion of poloxamers brought about a reduction in the ZP from ∼−20 mV (PP NPs) to ∼−31 mV (P NPs), which is crucial for their dimensional stability in suspension. In contrast, the presence of SLB within the NPs did not affect ZP values (Table 2) (*p* < 0.05) [16,34].

The encapsulation efficiencies of the SLBs were 84.8% and 93.9%, with actual loadings of 4.24 mg and 9.39 mg of SLB per 100 mg of polymer for PP-SLB5 and PP-SLB10 NPs, respectively. Furthermore, the drug loading percentages for the SLBs were found to be 1.61% for PP-SLB5 and 1.20% for PP-SLB10. The hydrophobic nature of SLB may be responsible for its high encapsulation efficiency. Interestingly, the increase in the entrapment efficiency was not accompanied by significant changes in NP zeta potential, whereas it increased the NP size, polydispersity index, and yield (*p* < 0.05) (Table 2). Studies of stability in double-distilled water at 4 °C for 30 days and in complete RPMI-1640 medium at 37 °C for 72 h proved that PP-NPs possess a satisfactory dimensional stability (Table 3).

**Figure 3:**
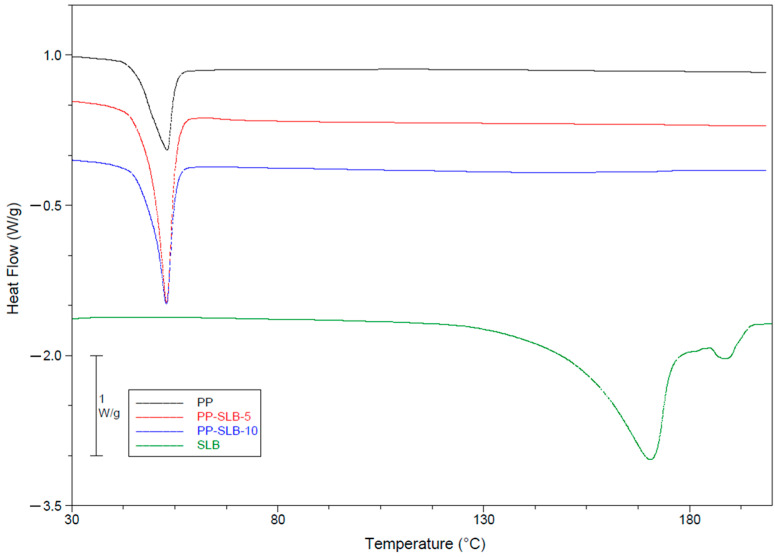
Thermograms of SLB and PLGA–poloxamer nanoparticles (PP NPs), with/without SLB. The exotherm is upwards.

The outcomes of thermal analyses are detailed in Figure 3 and Table 4. As can be observed from the thermograms depicted in Figure 3, all the NP formulations displayed an endothermic peak associated with the melting of poloxamers at 53.3 °C, while that of SLB presented a melting peak at 171.3 °C. The disappearance of the melting peak of SLB in drug-loaded NPs can be attributed to the loss of the crystalline forms of SLB. Therefore, DSC outcomes indicate that the active molecule is present as a molecular dispersion in the NPs [20,35].

### 3.2. In Vitro Drug Release Kinetics

**Figure 4:**
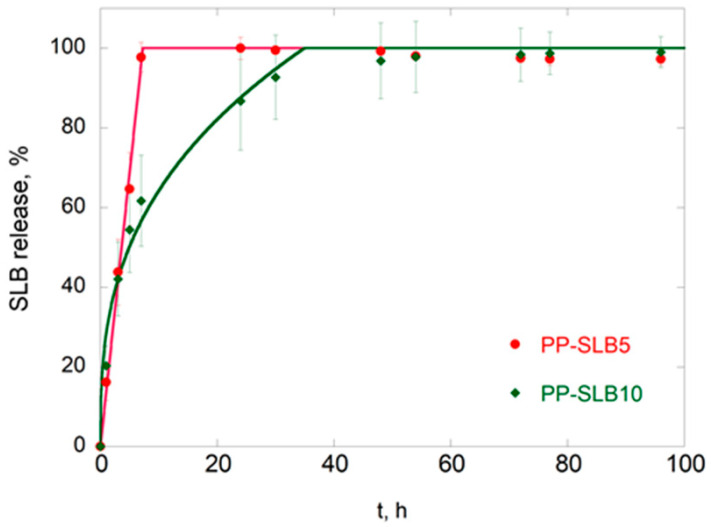
In vitro release profiles of SLB from PP-SLB5 and PP-SLB10 of NPs in PBS:DMF (9:1) at 37 °C. Solid lines represent best-fit simulations.

In vitro release curves of SLB from PP-SLB5 and PP-SLB10 NPs are shown in Figure 4. The release profiles were reproducible, thus showing the ability of PP-NPs to control and sustain SLB release. A significant burst effect was detected, in which approximately 97% and 73% of the SLB was released by PP-SLB5 and PP-SLB10 in 24 h, respectively, followed by a slower release phase. The release was completed within approximately 5 days.

Release data were fitted to Equations (2)–(5) to identify the conventional mathematical model that best describes SLB release and to verify whether different SLB loadings alter the release mechanism. Zero-order kinetics refer to a constant drug release rate over time. In contrast, the first-order model describes the release rate depending on the concentration of the remaining drug. The Higuchi equation models drug release as proportional to the square root of time. Finally, the semiempirical Korsmeyer–Peppas model describes drug release primarily dictated by diffusion, possibly coupled with other mechanisms that facilitate it [36]. Specifically, when applying the Korsmeyer–Peppas model to spherical particles, a value of *n* ≤ 0.43 indicates pure Fickian diffusion, while when 0.43 < *n* < 0.85, the release mechanism is considered anomalous diffusion, in which Fickian transport is associated with other mechanisms such as swelling and/or degradation [37,38].

The values of the fitting parameters used in the simulations are listed in Table 5.

The observation of R^2^ values indicates that for the PP-SLB 5 NPs, the best-fit release curves were obtained with the zero-order model (R^2^ = 0.997), while the Korsmeyer–Peppas equation better describes the release from the PP-SLB 10 NPs (R^2^ = 0.982). This indicates that at the lowest SLB loading, the driving force of release is constant over time; hence, desorption of the active molecule from the NP surface or nanoporosities prevails. In contrast, for PP-SLB 10 NPs, an n value of 0.307 indicates that diffusion is the primary mechanism governing SLB release from PLGA-based NPs within the experimental time frame, with negligible contributions from other mechanisms such as NP degradation [39]. This finding implies that SLB is also located in the inner regions of the NPs and clearly indicates that PLGA degradation occurs over a longer period than SLB release.

In general, drug release from polyester-based biodegradable devices in an aqueous environment is dictated by a complex diffusion–degradation mechanism triggered by water diffusion into the polymeric matrix [20,37,40]. PLGA degradation consists of the hydrolytic cleavage of ester bonds in the polymer backbone, which generates acidic degradation products. If these products accumulate in the polymeric matrix, they accelerate device degradation, thus establishing first-order autocatalytic degradation [41,42,43]. Once solubilized, the encapsulated molecule diffuses outwards through the interconnected pores of the polymer matrix [37]. This phenomenon has been observed in micrometric devices, for which it is necessary to use high concentrations of polymers in the organic phase (10–24% *w*/*v* in the organic phase) [37,40,44]. This favors the trapping of degradation products and therefore autocatalysis. In contrast, at the nanometric scale, polymer concentrations of approximately 2–4% *w*/*v* are used, leading to a much higher porosity, with a reduced possibility of degradation product accumulation within the NPs. Therefore, although the very high surface area of NPs rapidly triggers degradation, autocatalysis does not occur to a significant extent. Overall, NPs need a prolonged time for complete degradation, and other formulation strategies would be beneficial for optimizing and further sustaining drug release. In any case, SLB-PP5 formulation was selected to test the effectiveness of nanoencapsulation in promoting the bioactivity of SLB, since in a previous paper we had demonstrated that a mean size ≤ 100 nm strongly promotes NP cell internalization [45].

### 3.3. In Vitro Bioactivity of PP-SLB Nanoparticles

**Figure 5:**
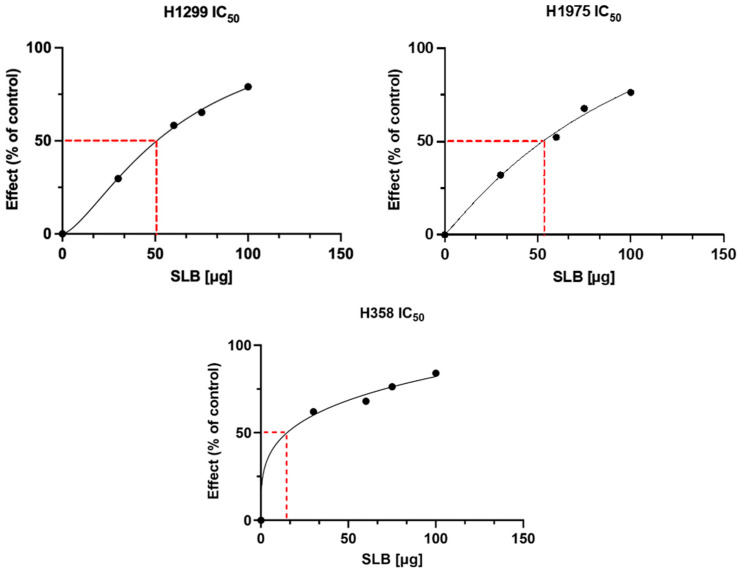
Analysis of the IC50 values. All cell lines were treated with increasing doses (30, 60, 75, 100 µg/mL) of SLB for 24 h.

The bioactivity of PP-SLB was tested in vitro on three different human lung cancer cell lines that differ in *p53* expression: H1299 [46] and H358 are *p53*-deficient [47], while H1975 expresses wild-type *p53* [48]. These cells were selected since NPs based on a PLGA–poloxamer blend showed a tropism toward lung tissue [20]. Preliminarily, to study in vitro cytotoxicity, cells were treated with increasing concentrations of SLB for 24 h, after which cell viability was measured. The results of cell cytotoxicity tests are reported in Figure 5. The IC_50_ was 54.70 ± 2 µg/mL for H1299 cells, 55.43 ± 2 µg/mL for H1975 cells, and 11.43 ± 2 µg/mL for H358 cells, showing that this latter cell line is the most sensitive (Figure 5).

**Figure 6:**
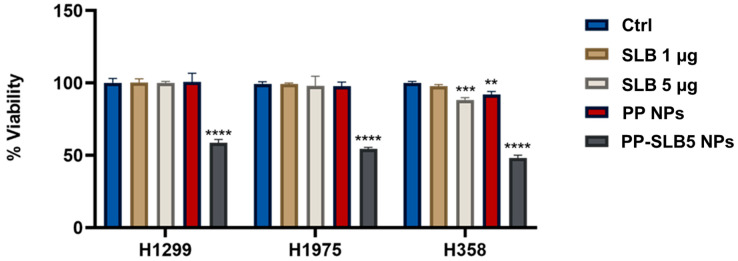
Efficacy of the PP-SLB5 NPs compared to the same amount of the free drug. Untreated cells and cells treated with unloaded NPs were used as controls (Ctrl, PP NPs). The data are presented as the mean ± standard deviation and represent at least three independent experiments (*n* = 3). ** *p* < 0.0001 NPs PP vs. control;; *** *p* < 0.0001 SLB 5 µg vs. control; **** *p* < 0.0001 vs. control.

Subsequently, the bioactivity of PP-SLB5 NPs was assessed from cytotoxicity tests against the three cell lines. The PP-SLB5 formulation, selected for its smaller particle size and rapid SLB release, was compared to the equivalent concentrations of free SLB. It must be underlined that, following filtration, the actual concentration of SLB in the NP suspension was found to be 2.53 µg/mL. The results of these experiments are shown in Figure 6.

As shown in Figure 6, PP-SLB5 NPs displayed a cytotoxic effect around the IC50 of all cell lines tested, while the same concentration of the free drug produced no detectable effect. In more detail, NPs induced cell death around 50% at an equivalent SLB concentration as low as 2.53 µg/mL, while the free drug caused no effect even at higher concentrations (5 µg/mL). Thus, these findings show that the cytotoxicity of SLB against human lung tumor cells was promoted by >10-fold. This result is particularly promising considering that the produced NPs displayed a tropism for the lung in vivo [20].

These outcomes are in line with the literature findings, which show that a significant amount of research interest has been devoted to testing the synergistic effect of SLB with currently used chemotherapeutics and improve its bioavailability profile [7,49,50]. In detail, in the field of nanomedicine, SLB has been tested in combination with glycyrrhizic acid and loaded in PEGylated nanoliposomes, which showed a >10-fold increase in SLB bioactivity against human hepatocellular carcinoma cells [51]. In another report, Kuen et al. demonstrated that SLB loaded in chitosan-based NPs displayed enhanced cytotoxicity against the A549 lung tumor cell line [52]. To the best of our knowledge, this is the first time that PLGA–poloxamer NPs have been tested for SLB delivery and bioactivity toward the human lung cancer cells used in this study.

## 4. Conclusions

The formulated NPs proved to be safe both in vitro and in vivo, as they demonstrated preferential accumulation in the lungs [20]. Furthermore, PP NPs can increase the bioactivity of SLBs, as shown by in vitro experiments on lung cancer cells. Despite the promising results obtained in this study, the proposed formulations still present quick release, and it may be desirable to sustain them for longer periods of time in specific therapies. However, the results of the mathematical model comparison demonstrated that by varying the amount of drug loaded into the NPs, it is possible to exploit different release mechanisms. Specifically, in the case of the formulations with the highest SLBs, the release mechanism is diffusive. The diffusion of SLBs is likely to occur through the polymeric matrix and nanoporosities of the device. This suggests that by narrowing the nanoporosities of the NPs through proper formulation changes, it is in principle possible to sustain SLB release. This goal can be achieved by varying the molecular weight and/or concentration of PLGA in the organic phase and/or by further increasing the drug loading. Future studies will therefore focus on optimizing the formulation to prolong SLB release.

## Figures and Tables

**Table 1:** Composition and acronyms of the different concentrations of the NP formulations.

Sample Acronym	Polymer Concentration in the Organic Phase Oil % *w*/*v*				Polox in the Aqueous Phase % *w*/*v*
	PLGA%	F68%	F127%	F68%	F127%
	(*w*/*v*)	(*w*/*v*)	(*w*/*v*)	(*w*/*v*)	(*w*/*v*)
P	2			0.0175	0.0175
PP	1	0.5	0.5	0.0175	0.0175
PP-SLB5	1	0.5	0.5	0.0175	0.0175
PP-SLB10	1	0.5	0.5	0.0175	0.0175

**Table 2:** NP size, polydispersity index (PDI), and zeta potential in distilled water at 4 °C. The entrapment efficiency, drug loading, yield values, and standard deviations were calculated from the last three independent experiments.

Formulation	Particle Mean Diameter (nm)	Polydispersity Index (PDI)	Zeta Potential (mV)	Entrapment Efficiency (%)	Drug Loading (%)	Yield (%)
P	153 ± 1.6 *	0.140 ± 0.01	−31.4 ± 2.0 *			
PP	89 ± 0.6 *^#$^	0.132 ± 0.01 ^$^	−19.2 ± 2.0 *	-		57.02 ± 0.4 ^#$^
PP-SLB5	98 ± 2.2 ^#&^	0.212 ± 0.03 ^&^	−20.9 ± 0.4	84.8 ± 3.0	1.61 ± 1.8	74.3 ± 2.5 ^#^
PP-SLB10	124 ± 2.0 ^$&^	0.302 ± 0.06 ^$&^	−21 ± 2.0	93.9 ± 2.1	1.20 ± 0.1	72.8 ± 1.8 ^$^

* *p* < 0.05 P vs. PP; ^#^ *p* < 0.05 PP vs. PP-SLB5; ^$^ *p* < 0.05 PP vs. PP-SLB10; ^&^ *p* < 0.05 PP-SLB5 vs. PP-SLB10.

**Table 3:** Time evolution of mean NP diameters in water at 4 °C and in cell culture medium (RPMI) at 37 °C for different NP formulations loaded with SLB. All the results are expressed as the mean value ± the standard deviation of three independent runs.

Formulation	NP Diameters in Water 4 °C [nm]				NP Diameters in RPMI 37 °C [nm]		
	Day 0	Day 10	Day 20	Day 30	Day 1	Day 2	Day 3
P	153 ± 1.6	154 ± 0.2	160 ± 1.1	182 ± 5.4	166 ± 9.4	168 ± 2.5	191 ± 1.5
PP	89 ± 0.6	92 ± 0.4	89 ± 0.6	90 ± 1.0	85 ± 0.5	84 ± 0.8	84 ± 0.3
PP-SLB5	98 ± 2.2	156 ± 3.3	153 ± 2.3	156 ± 1.9	153 ± 4.0	160 ± 2.0	164 ± 1.2
PP-SLB10	124 ± 2.9	185 ± 1.5	163 ± 1.0	186 ± 4.7	130 ± 2.5	197 ± 1.8	198 ± 15

**Table 4:** Melting temperature (Tm), onset temperature (Tonset), and enthalpy change (ΔH°m) of the unloaded and SLB-loaded PLGA–poloxamer nanoparticles (PP NPs).

Sample	ΔH° (J/g)	T_onset_ (°C)	T_m_ (°C)
PP	28.6 ± 2.5	47.0 ± 1.9	53.3 ± 0.2
PP-SLB5	52.8 ± 3.7	46.9 ± 2.6	52.3 ± 1.1
PP-SLB10	40.7 ± 1.2	49.4 ± 1.0	53.3 ± 0.5
SLB	172.3 ± 10.4	152.6 ± 2.2	171.3 ± 1.0

**Table 5:** Fitting parameters used in the simulations.

		SLB 5		SLB 10	
Model	Parameter		R^2^		R^2^
Zero order	*k*_0_ [h^−1^]	0.133	0.997	0.082	0.949
First order	*k*_1_ [h^−1^]	0.227	0.937	0.084	0.971
Higuchi	*k_H_* [h^−0.5^]	0.300	0.942	0.158	0.977
Korsemeyer–Peppas	*k_KP_* [h^−n^]	0.151	0.935	0.259	0.982
	*n*	0.927		0307	

## Data Availability

The original contributions presented in the study are included in the article, further inquiries can be directed to the corresponding authors.
